# Presence of Meaning in Life in Older Men and Women: The Role of Dimensions of Frailty and Social Support

**DOI:** 10.3389/fpsyg.2021.730724

**Published:** 2021-09-03

**Authors:** Nadezhda Golovchanova, Christiana Owiredua, Katja Boersma, Henrik Andershed, Karin Hellfeldt

**Affiliations:** School of Law, Psychology and Social Work, Örebro University, Örebro, Sweden

**Keywords:** presence of meaning, meaning in life, older adults, frailty, social support, gender differences

## Abstract

Presence of meaning in life is an important component of eudemonic wellbeing while aging. While subjective health and interpersonal relationships are among important sources of meaning for older adults, less research has explored the gender differences in the potential contribution of these sources to the presence of meaning in late life. The current study aims to examine the associations of frailty dimensions (daily activities, health problems, and psychosocial functioning) and social support with the presence of meaning in late life, and whether these associations differ for older men and women. The study employs the data from the 65+ and Safe Study – a cross-sectional survey of residents of senior apartments. The data were collected in 2019 in a mid-sized Swedish municipality (*N*=618; age range from 64 to 106years, 60.5% female). Results showed significant associations of health problems, psychosocial functioning, and social support with the presence of meaning in life. Further, the results demonstrated no statistically significant gender differences in the associations between frailty dimensions, social support, and presence of meaning. However, since the interaction between health problems and gender approached statistical significance, this association was further explored indicating a more detrimental role of health problems in relation to the presence of meaning in life among older men than among older women. Overall, the study highlights the importance of physical and psychosocial health and social support for the presence of meaning in life among older adults and warrants further research on possible gender differences in the relation between health problems and meaning in late life.

## Introduction

Maintaining meaning in life is an important part of eudemonic wellbeing for older adults (Steptoe et al., 2015; Steverink, 2019). Meaning and purpose in life are recognized among psychological outcomes in the current understanding of successful ageing (Fernandez-Ballesteros, 2019). Recently, the presence of meaning in late life has been gaining research attention because it has been positively associated with life satisfaction, happiness, and positive affect (Steger et al., 2009) and negatively associated with depressive feelings (Van der Heyden et al., 2015; Volkert et al., 2019). Considering frequent losses and challenges that often accompany the aging process (Kuin and Westerhof, 2017), it is especially important to understand the role of factors hindering or maintaining meaning in life into old age. Such understanding is important for identifying those at most risk for meaninglessness while aging and improving the targeting of meaning enhancing interventions (e.g., Westerhof et al., 2004).

The presence of meaning in life can be defined as “the extent to which people comprehend, make sense of, or see significance in their lives, accompanied by the degree to which they perceive themselves to have a purpose, mission, or overarching aim in life” (Steger et al., 2009, p. 43). Thus, the presence of meaning does not refer to the global or ultimate meaning of a human life, but rather reflects a personal perception of the degree to which one’s life is meaningful. In light of the developmental tasks of aging related to developing an integrative and transcending view over one’s whole life, such meaningfulness is especially important in late life (Krause, 2012).

However, older age is a life period marked with high heterogeneity (Diehl and Wahl, 2020), especially in health aspects and overall functioning. Considering that health aspects were previously associated with meaning in life (Roepke et al., 2013; Czekierda et al., 2017), the variation in health is among important factors explaining the differences in meaningfulness in late life. For instance, the number of physical illnesses has been shown to be negatively associated with meaning in life in older adults (Volkert et al., 2019). A more complex health indicator specific for late life is frailty which is referred to as a multidimensional construct that reflects declines related to multiple functioning domains, such as being able to carry out daily tasks, having specific health problems, or psycho-social complaints (Schuurmans et al., 2004; Bielderman et al., 2013). Although a general negative association between frailty and subjective wellbeing has been reported (Steverink, 2019), less is known about associations of specific frailty dimensions with the presence of meaning. Theoretically, given the multidimensionality of frailty, the complex interplay among different frailty dimensions needs consideration in relation to older adults (Markle-Reid and Browne, 2003). Thus, it is important to understand whether losses in certain dimensions are stronger and independently associated with the presence of meaning. Such knowledge would enable both researchers and health care practitioners to improve recognizing those older adults who might perceive their life as less meaningful, and, consequently, experience lower eudemonic wellbeing. Also, since studies indicate gender differences in different frailty dimensions (for review, Collard et al., 2012), it is also important to extend our understanding on whether these potential associations differ for men and women, a knowledge gap this study aims to address.

Further, relations with others have been previously outlined as the central source of meaning across the life span (Glaw et al., 2017). In late life, the presence of meaning was positively associated with a number of significant social contacts in older adults (Volkert et al., 2019), with the quality of interpersonal relationships (Dewitte et al., 2019) and with aspects of social support (Krause, 2007). The term social support generally refers to different kinds of supportive social relations or interactions that increase or promote an individual’s wellbeing (Cohen et al., 2000). According to the main effect model on social support, social support is beneficial for an individual’s wellbeing since stable supportive network gives the individual a sense of belonging and security. In late life, the social environment and social relations have been argued to be an important source of meaning (O'Donnell et al., 2014; Duppen et al., 2019). In the current study, we further explore the role of social support in the presence of meaning of older men and women.

Moreover, considering previously reported gender differences in aging trajectories (Li et al., 2020), gender emerges as a potentially important factor in relation to factors associated with the presence of meaning while aging. Women display higher prevalence of frailty (Freitag and Schmidt, 2016; Gordon et al., 2017) and lower psychological health (Pinquart, 2002), and are more likely to become widows (Calasanti, 2010). Because these factors are closely associated with previously outlined sources of meaning (i.e., health and social relationships), deficits in these sources might lead to women experiencing lower presence of meaning compared to men in late life. Research showed that women above 70years old experience less meaning in life compared to men (Pinquart, 2002). However, other studies showed no gender differences in the presence of meaning in late life (Krause, 2007), or, in contrast, lower presence of meaning experienced by older men (Volkert et al., 2019). Thus, more research is needed to understand the role of gender in the presence of meaning in life and in associations between frailty dimensions, social support, and the presence of meaning.

### The Current Study

The current study aimed to investigate whether frailty dimensions (i.e., daily activities, health problems, and psychosocial functioning) and social support were associated with the presence of meaning in advanced age. Since less is known about potential gender differences in these associations, we addressed this research gap by exploring whether these associations differed for older men and women. Because autonomy (Hupkens et al., 2021), subjective health (Czekierda et al., 2017), and social connectedness (Glaw et al., 2017) were previously reported as important sources of meaning, we hypothesized that higher frailty scores in the respective dimensions of daily activities, health problems, and psychosocial functioning would be independently negatively associated with the presence of meaning in older adults. Further, in line with previous findings (Pinquart, 2002; Krause, 2007; Volkert et al., 2019), we expected social support to be positively associated with presence of meaning. Additionally, we explored the possible gender differences in these associations.

## Materials and Methods

### Sample and Procedure

The current study employs the data of the 65+ and Safe Study – a cross-sectional survey study of residents of senior apartments carried out in 2019 in a Swedish municipality. Such apartments are a form of senior housing in Sweden, available for people 65years and older. These apartments differ from nursing home or assistant living settings. Most of the apartment buildings are adapted for older adults and are equipped with automatic door openers, elevators, etc. Senior apartments are located in both urban and rural areas of the municipality, differ in size, and are situated in neighborhoods with diverse socioeconomic status. Potential participants were contacted by written mail with an invitation to complete the survey questionnaire in paper form or *via* a Web site. These initial survey letters were followed by reminder letters and reminder phone calls administered within the following weeks of the data collection. Inclusion criteria were: (1) becoming 65years old in the year of 2019, or older; (2) residing in a senior apartment, and (3) the absence of severe cognitive impairment. The project was approved by the Swedish Ethical Review Agency (Dnr: 2019-02248). All participants of the 65+ and Safe Study provided a written informed consent.

The study sample comprised of 622 participants indicating the response rate of 49.5%. The power calculation was performed prior to the data collection indicating that considering the power of 80% and *p*<0.05 in a two-tailed distribution, the sample of at least 194 participants was required for expected correlation coefficients of *r*=0.2. Based on the previous research which indicated that associations of the presence of meaning with self-rated health and social support tend to be stronger than *r*=0.2, the obtained sample was considered sufficient for performing the study. There were no significant gender differences between responders and non-responders [*χ*^2^ (2, *N*=1,237)=1.17, *p*=0.56]. However, responders were on average younger (*M*=77.6, *SD*=7.2) compared to those who did not respond [*M*=79.1, *SD*=8.1; *t* (1220)=−3.41, *p*=0.001]. Out of the 622 respondents, four people were missing data on all study variables (both predictors and outcome variables) except for gender and age; hence, these were excluded from further analysis. This resulted in a sample size of 618 participants. The average age of the study participants was 77.6years (*SD*=7.2; age range 64–106); 60.5% were women. In this sample, 68.4% reported having education similar to a high school degree or lower, 36.7% were married, 7.8% lived together with a partner, 2.4% lived separately with a partner, 15.9% were divorced, 9.7% were single, and 26.1% were widowed. Additionally, 23.3% received assistance with daily tasks (e.g., with preparing meals and grocery shopping).

### Measurements

#### Presence of Meaning in Life

The presence of meaning was assessed with the “Presence of Meaning” subscale of the Meaning in Life Questionnaire (Steger et al., 2006). The subscale consists of five items rated by the participants on a 7-point Likert scale from 1 (Absolutely untrue) to 7 (Absolutely true). Examples of items are “I understand my life’s meaning” and “My life has a clear sense of purpose.” The Presence of Meaning variable used in this study was calculated using the sum score of the five items. The subscale demonstrated good reliability in our sample (Cronbach’s alpha 0.80).

#### Frailty

Frailty was assessed with the Groningen Frailty Indicator (GFI; Steverink et al., 2001). This is a multidimensional screening instrument which contains 15 items that assess physical, cognitive, social, and psychosocial dimensions of frailty. Eight items include “Yes” or “No,” and six items include “Yes,” “Sometimes,” and “No” as response categories, while one item is scored on a 10-point Likert scale. Responses on all items were coded according to the scale scoring [for exact coding of each item, please see Schuurmans et al. (2004)]. Higher scale score indicates higher level of frailty with the score of four and greater being indicative of frailty (Schuurmans et al., 2004). The scale showed good reliability in our sample [Kuder-Richardson (KR) 20=0.70].

In the current study, we examined three dimensions of frailty separately according to the previously demonstrated three-dimensional structure of the GFI (Bielderman et al., 2013). The Daily Activities subscale assessed the ability to carry out daily tasks (dressing, going to the toilet, shopping, and walking outdoors; items 1–4). The Health Problems subscale assessed the presence of specific health problems (e.g., vision, hearing, weight loss, and memory; items 5–10). The Psychosocial Functioning subscale assessed the presence of psycho-social complaints (e.g., experience of emptiness, feeling downhearted or sad, and feeling nervous or anxious; items 11–15). Values of the KR 20 for the Daily Activities, Health Problems, and Psychosocial functioning were 0.68, 0.40, and 0.78, respectively. Deleting any of the items did not improve reliability of the subscales. Poor reliability of the Health Problems subscale was previously observed in a sample of older adults and can be explained by the substantial heterogeneity of health problems covered by the subscale (Bielderman et al., 2013). Despite this limitation, we considered it possible using the subscale as indicative of the presence of health problems in our sample.

#### Social Support

Social support was assessed with the Multidimensional Scale of Perceived Social Support (Zimet et al., 1988). The scale consists of 12 items which assess perceived social support from the significant other, family, and friends. Each item was scored on a 7-point Likert scale from 1 (Very strongly disagree) to 7 (Very strongly agree). Examples of items are “There is a special person who is around when I am in need,” “I can talk about my problems with my family,” and “I have friends with whom I can share my joys and sorrows.” To create the variable used in the study, the sum of the twelve items was calculated. The scale showed excellent reliability in our sample (Cronbach’s alpha 0.94).

### Data Preparation and Analytic Procedure

All data analysis was conducted using the IBM SPSS 27. Before commencing with data analysis, missing data analysis was performed to estimate the level of missing data as well as the nature of the missingness with regard to it randomness. The diagnostics of the pattern of missingness in data was conducted with results indicating that 6% of all data values were missing with 72% of the participants with complete data on all variables. Further, the Little’s MCAR test indicated that the data were not missing completely at random [MNAR; *χ*^2^ (87)=131, *p*<0.01]. Assuming missing data to be missing at random, listwise deletion may lead to biased estimate; hence, multiple imputation with five iterations was used to deal with missingness in the data (Jakobsen et al., 2017). This method is suggested to result in unbiased estimate with the proportion of data missing less than 40% (Jakobsen et al., 2017). Further, to rule out the potential impact data not missing completely at random, sensitivity analysis was performed as a control by running the analysis on the initial data with missing values, with no noteworthy differences in the direction of associations among the variables observed.

Descriptive analysis consisted of calculating the means, standard deviations, percentage scores when appropriate, and correlation coefficients between the study variables. The main analysis included multiple regression models with the presence of meaning as outcome. In the first model, frailty dimensions (daily activities, health problems, and psychosocial functioning) and social support were entered while controlling for age and gender. The second model included interaction terms of each of the frailty dimensions and social support with gender, in addition to the independent variables included in the first model. In order to reduce multicollinearity, interaction terms were created with mean-centered independent variables (Hayes, 2018). No substantial deviation from normal distribution in the outcome variable (presence of meaning) was detected (Skewness=−0.277, *SE*=0.098; Kurtosis=0.004, *SE*=0.196). Multicollinearity diagnostics revealed no multicollinearity problems in the first model and acceptable multicollinearity in the second model (all tolerance values above 0.2 and all VIF values below 5). PROCESS v3.5 was used to visualize possible gender differences.

## Results

### Descriptive Statistics

Table 1 presents the descriptive statistics of study variables. There were no gender differences in the presence of meaning and the frailty dimension daily activities. However, gender differences were found in the frailty dimension health problems and psychosocial problems and in social support. Table 2 presents the correlation coefficients between the study variables. The presence of meaning was significantly associated to age as well as with frailty dimensions (Daily activities, Health problems, and Psychosocial functioning) and social support. All associations were significant except for those between age and psychosocial functioning subscale, age and social support, gender and presence of meaning, gender and daily activities, and daily activities subscale and social support (Table 2).

**Table 1:** Descriptive statistics and *t*-test results for gender differences for the study variables.

Variable	Overall sample *M* (*SD*)	Men *M* (*SD*)	Women *M* (*SD*)	*t*	Value of *p*
Presence of Meaning	24.39 (5.71)	23.94 (6.12)	24.68 (5.42)	−1.532	0.126
Age	77.56 (7.20)	77.02 (6.94)	77.90 (7.36)	−1.485	0.138
Daily Activities	0.20 (0.58)	0.15 (0.53)	0.23 (0.61)	−1.751	0.080
Health Problems	2.10 (1.26)	2.30 (1.28)	1.97 (1.23)	3.283	**0.001** ^**^
Psychosocial Functioning	1.99 (1.71)	1.74 (1.64)	2.15 (1.74)	−2.928	**0.004** ^**^
Social Support	64.72 (14.55)	62.75 (16.59)	66.01 (12.91)	−2.604	**0.010** ^*^

*M*, mean; *SD*, standard deviation.

* *p*<0.05 and

** *p*<0.01.

**Table 2:** Correlations among the study variables.

S. No.		1	2	3	4	5	6	7
1.	Age	–						
2.	Gender^a^	0.06	–					
3.	Presence of Meaning	−0.14^**^	0.06	–				
4.	Daily Activities	0.24^**^	0.07	−0.09^*^	–			
5.	Health Problems	0.30^**^	−0.13^**^	−0.25^**^	0.29^**^	–		
6.	Psychosocial Functioning	0.03	0.12^**^	−0.30^**^	0.08^*^	0.29^**^	–	
7.	Social Support	0.01	0.11^**^	0.49^**^	−0.02	−0.09^*^	−0.29^**^	–

a 0 = men, 1 = women

Listwise *N*=618.

* *p*<0.05 and

** *p*<0.01

### Multivariate Associations Between Frailty Dimensions, Social Support, and Presence of Meaning

To test whether the frailty dimensions (Daily activities, Health problems, and Psychosocial functioning) and social support contribute to significant variance in presence of meaning in addition to age and gender, a multiple regression analysis was performed (Table 3; Model 1). The model was statistically significant [*F* (6, 611)=45.05, *p*<0.01] explaining 30% of variance in the presence of meaning (as indicated by the adjusted *R*^2^). Specifically, age, health problems, psychosocial functioning, and social support explained unique variance in the presence of meaning. Higher age and higher frailty in the health and psychosocial dimensions were independently negatively associated with the presence of meaning, while social support was independently positively related to the presence of meaning.

**Table 3:** Multiple regression analyses with the presence of meaning as dependent variable.

Variable	*B*	*SEB*	*β*	95% CI		Value of *p*
				*LL*	*UL*	
***Model 1***						
Age	−0.08	0.03	−0.10	−0.13	−0.02	0.007^**^
Gender^a^	0.22	0.41	0.02	−0.59	0.03	0.598
Daily Activities	−0.13	0.36	−0.01	−0.83	0.56	0.709
Health Problems	−0.61	0.18	−0.14	−0.96	−0.27	0.001^**^
Psychosocial Functioning	−0.43	0.13	−0.13	−0.67	−0.18	0.001^**^
Social Support	0.17	0.01	0.44	0.15	0.20	< 0.001^**^
Adjusted *R*^2^ =0.30						
***Model 2***						
Age	−0.08	0.03	−0.11	−0.14	−0.03	0.004^**^
Gender^a^	0.18	0.42	0.02	−0.63	1.00	0.664
Daily Activities	−0.58	0.61	−0.06	−1.77	0.61	0.340
Daily Activities×Gender	0.65	0.75	0.05	−0.81	2.12	0.383
Health Problems	−0.98	0.27	−0.22	−1.51	−0.45	< 0.001^**^
Health Problems×Gender	0.64	0.35	0.11	−0.04	1.32	0.066
Psychosocial Functioning	−0.31	0.21	−0.09	−0.73	0.11	0.145
Psychosocial Functioning×Gender	−0.18	0.26	−0.04	−0.69	0.34	0.507
Social Support	0.17	0.02	0.44	0.13	0.21	<0.001^**^
Social Support×Gender	0.01	0.03	0.01	−0.05	0.06	0.805
Adjusted *R*^2^=0.30						

a 0 = men; 1 = women.

*N*=618; CI, confidence interval; *LL*, lower limit; and *UL*, upper limit. ^*^p<0.05 and

** *p*<0.01.

### Gender Differences in the Associations Between Frailty Dimensions, Social Support, and Presence of Meaning

**Figure 1:**
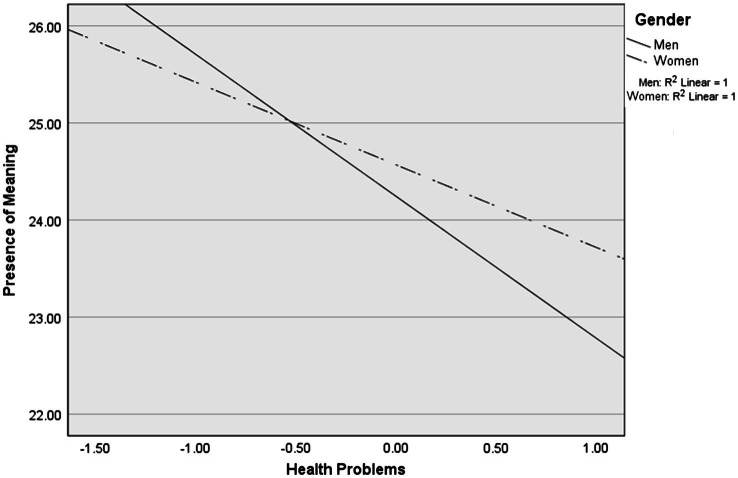
Gender differences in the association between health problems and the presence of meaning.

To explore possible gender differences in associations between the frailty dimensions (Daily activities, Health problems, and Psychosocial functioning), social support and the presence of meaning, a multiple regression model with interactions between the independent variables and gender, in addition to the independent variables included in the first model, was performed (Table 3; Model 2). The model was statistically significant [*F* (10, 607)=27.67, *p*<0.01] and explained 30% of variance in the presence of meaning (as indicated by the adjusted *R*^2^). The results showed that none of the interaction term variables reached statistical significance at *p*<0.05. However, because the interaction of Health problems×Gender approached statistical significance (*p*=0.07), gender differences in the association of health problems and the presence of meaning were further explored. As shown in Figure 1, this analysis indicated that, with increase in health problems, men showed stronger decline in the presence of meaning compared to women. Conditional effects were statistically significant (*p*<0.01) for both genders.

## Discussion

The current study investigated the presence of meaning and its associations with frailty dimensions and social support, as well as possible gender differences in these associations in a sample of senior apartment residents. In line with our hypothesis, health and psychosocial problems were independently negatively associated with the presence of meaning in the total sample. This result highlights the importance of both health-related and psychosocial frailty as factors independently related to less presence of meaning in life. Considering that 90% of frail individuals report difficulties in their psychosocial functioning (Bielderman et al., 2013), it is especially important to monitor the association of this frailty dimension with meaning in life. In contrast with our hypothesis, the daily activities dimension was not significantly associated with presence of meaning. Possibly, considering that a substantial amount of the residents of senior apartments receive assistance with daily tasks at home, such help might enable these older adults to maintain autonomy and to continue engaging with important sources of meaning. This is in line with a recent finding that older adults receiving care at home perceive sufficient autonomy for maintaining meaning and report a broad variety of meaning in life sources (Hupkens et al., 2021). Additionally, older study participants reported significantly less presence of meaning which calls special attention to those in the highest age as experiencing less meaning in life. As observed by Pinquart (2002), this could be explained by the losses in the domains that constitute important sources of meaning for older adults (e.g., health, social relationships, and the ability to participate in activities) which become more frequent with higher age.

Further, perceiving social support from a significant other, friends, and family was associated with more presence of meaning in life. This finding underscores the central role of social context in acquiring meaning (Kuin and Westerhof, 2017) and articulates the crucial role of social support in maintaining the presence of meaning in late life. Prioritizing emotionally significant contact with others over less fulfilling connections as a way to maintain higher levels of wellbeing has been suggested by the socioemotional selectivity theory (Carstensen et al., 2003). Striving for redefinition and more selectivity in relationships with others is articulated as a sign of the gerotranscendence process which is a hallmark of late life (Tornstam, 2005) and which is associated with meaning in life in older adults (Braam et al., 2006). The study findings endorse these theoretical propositions by showing that those older adults who have available sources of social support in their social networks experience their lives as more meaningful. The findings might also suggest that older adults lacking social support might benefit from interventions directed at creating opportunities for developing close contact with others to enhance the presence of meaning in their lives. Further research might explore the relationships with family members (partner, children, and grandchildren), friends, or other people in relation to meaningfulness.

Regarding gender differences, we observed no significant differences in levels of presence of meaning between men and women in our sample. Further, the study demonstrated no gender differences in the associations of daily activities, psychosocial functioning, and social support with presence of meaning in late life. However, the near significant interaction (*p*=0.07) between health problems and gender indicated that the association between declining health and meaning in life might be moderated by gender. Tentatively, health problems may be more detrimental for men than for women in influencing presence of meaning. One explanation for this finding could be that significance of different sources of meaning might differ for men and women in later life. Although some research concluded that gender is not a key factor in preferred sources of meaning (Bar-Tur et al., 2001; Reker and Woo, 2011), another study found that leisure time was more likely to be a source of meaning for older men compared to older women (Grouden and Jose, 2014). Experiencing health problems could create a substantial barrier for participating in leisure time activities, which could be more important for men. Therefore, although health problems are significantly related to presence of meaning for both men and women, this association might be stronger for older men because of its interference with an important source of meaning.

Taken together, these findings indicate that problems with psychosocial functioning and lack of social support are of equal importance for men and women as predictors of meaning in life while aging. Experiencing health problems, however, might be differentially related to meaning in life for men and women while aging. Specifically, men might be at higher risk for experiencing their life as less meaningful when experiencing substantial health problems. However, these findings are exploratory and warrant replication in other samples of older adults.

### Strengths and Limitations

The main strength of the present study includes the wide age range (64–106years old), allowing to investigate the presence of meaning across the aging span. The sufficient sample size allowed to study the several factors associated with presence of meaning, including the potential differences between older men and women. Moreover, the study examined the distinct dimensions of frailty which is a multidimensional age-relevant health indicator, social support, and explored whether gender differences existed in the associations between these factors and presence of meaning.

Several limitations of the study should be mentioned. First, some measurement questions should be considered. Because health problems subscale of GFI showed low reliability, which can most likely be attributed to a broad diversity of health complaints that its questions tap into, it might be beneficial to replicate our findings in other samples to ensure robustness of the results. Further research might explore the associations of specific health problems (e.g., vision, hearing problems, and overall fitness) with the presence of meaning. Additionally, further research might consider more frailty dimensions (e.g., environmental frailty and cognitive frailty; De Donder et al., 2019) and their associations with presence of meaning in late life. Further, because the current study included the measure of emotional social support only, further research might explore the role of informational, instrumental, or other forms of social support (Uchino et al., 2016) in relation to presence of meaning. Secondly, the study sample included only older adults living in senior apartments. This suggests caution in generalizing the results of the study to the general population of older adults because the residents of senior apartments might differ from, for instance, homeowners or nursing home residents in their health, socioeconomic status, or life-style aspects. Additionally, because the age of application for senior apartments may vary between 55 and 65years in Sweden, some residences might host considerably younger adults. Moreover, responders of the survey were slightly younger compared to non-responders. Considering that the presence of meaning was lower for older individuals, more research specifically focused on those in the highest age is needed. Yet, considering that average level of presence of meaning in our study was within the range of those reported in previous studies on community dwelling older adults (e.g., Steger et al., 2009; Hofer et al., 2014; Van der Heyden et al., 2015), we suppose that these sample specifics do not have a detrimental impact on the main study outcome variable. Thirdly, because the study questionnaire was made available only in Swedish and in English, the sample excluded those prospective respondents who were not able to respond in either of these two languages.

## Conclusion

The current study examined the role of frailty dimensions and social support in the presence of meaning of older adults – residents of senior apartments. The results showed that experiencing less problems in health and psychosocial functioning dimensions of frailty significantly and independently contributed to the presence of meaning in late life. Thus, the findings contributed to the body of previous research which established associations between subjective health and meaning in late life. Moreover, the study showed that social support is an important resource for experiencing the presence of meaning in late life, thus endorsing the theoretical propositions of the importance of emotionally close relations for maintaining eudemonic wellbeing in late life. We observed no gender differences in the associations between daily activities and psychosocial functioning, as well as social support with the presence of meaning. However, the gender differences in the association between health problems and presence of meaning deserve further research attention as declining health might be more detrimental for loss of meaning for men compared to women. This is important to consider in the future research on meaning in life as well as for psychological interventions aimed at enhancing meaningfulness in older adults.

## Data Availability Statement

The datasets presented in this article are not readily available because of participant confidentiality. Requests to access the datasets should be directed to karin.hellfeldt@oru.se.

## Ethics Statement

The studies involving human participants were reviewed and approved by the Swedish Ethical Review Agency (Dnr: 2019–02248). The patients/participants provided their written informed consent to participate in this study.

## Author Contributions

NG, CO, KB, HA, and KH: conceptualization, methodology, and analytic plan. NG and CO: formal analysis and writing—original draft preparation. NG, HA, and KH: investigation, resources, project administration, and funding acquisition. NG, KB, HA, and KH: writing—review and editing. All authors have read and agreed to the published version of the manuscript.

## Conflict of Interest

The authors declare that the research was conducted in the absence of any commercial or financial relationships that could be construed as a potential conflict of interest. The handling editor declared a past co-authorship with one of the authors NG.

## Publisher’s Note

All claims expressed in this article are solely those of the authors and do not necessarily represent those of their affiliated organizations, or those of the publisher, the editors and the reviewers. Any product that may be evaluated in this article, or claim that may be made by its manufacturer, is not guaranteed or endorsed by the publisher.
